# Edge-Triggered Three-Dimensional Object Detection Using a LiDAR Ring

**DOI:** 10.3390/s24062005

**Published:** 2024-03-21

**Authors:** Eunji Song, Seyoung Jeong, Sung-Ho Hwang

**Affiliations:** Department of Mechanical Engineering, Sungkyunkwan University, 2066 Seobu-ro, Suwon 16419, Republic of Korea; h2ejsong@skku.edu (E.S.); nasy960@skku.edu (S.J.)

**Keywords:** autonomous driving, object detection, rule-based, 3D LiDAR, edge-triggered

## Abstract

Autonomous driving recognition technology that can quickly and accurately recognize even small objects must be developed in high-speed situations. This study proposes an object point extraction method using rule-based LiDAR ring data and edge triggers to increase both speed and performance. The LiDAR’s ring information is interpreted as a digital pulse to remove the ground, and object points are extracted by detecting discontinuous edges of the z value aligned with the ring ID and azimuth. A bounding box was simply created using DBSCAN and PCA to check recognition performance from the extracted object points. Verification of the results of removing the ground and extracting points through Ring Edge was conducted using SemanticKITTI and Waymo Open Dataset, and it was confirmed that both F1 scores were superior to RANSAC. In addition, extracting bounding boxes of objects also showed higher PDR index performance when verified in open datasets, virtual driving environments, and actual driving environments.

## 1. Introduction

For the advancement of autonomous driving LV3 and LV4 defined by [1], real-time high-performance recognition technology is required even at high speeds. Many companies have installed these LV3 self-driving technologies in their mass-produced cars in the form of self-driving on highways. In the case of high speed, if the logic execution delay time and recognition distance are not substantial, obstacles can quickly approach due to speed and become dangerous. Therefore, efforts are needed to increase the cognitive distance and speed up logic execution.

The deep learning method using the point cloud of LiDAR has steadily developed, and its performance has been advanced [2]. It recognizes objects as bounding boxes by learning datasets divided into three classes, mainly cars, pedestrians, and cyclists, with neural networks. Its performance depends on learning datasets and network structures. To improve the performance, studies that directly construct many object recognition datasets in various real environments have been conducted [3]. Recently, many researchers also have built a dataset in a virtual environment to expands the diversity of situations [4]. In addition, researchers have actively conducted to improve the structure of neural network models [5,6]. However, performance-based network architectures cannot be used in actual driving unless applied in real time. Also, regardless of real-time and recognition performance, learning relies on datasets, mainly on three classes (vehicle, pedestrian, cyclist). So, other objects that vehicles need to recognize must be correctly recognized.

To solve the limitations of the obstacle recognition type, on the contrary, there are also studies to learn the driving area of the vehicle using semantic segmentation [7]. However, semantic segmentation using the 3D point cloud has heavy data, so a model that satisfies both performance and real-time requirements has yet to be developed. According to the state of the art, in terms of performance alone, the best model [8]’s mIoU is 72.9. However, looking at the real-time models, the mIoU of the model [9] that satisfies the minimum reference level of 98fps in real time is 46.9. So, the model that satisfies both real-time and performance requirements still needs to be completed.

Most semantic segmentation models using a camera satisfy both real-time and performance requirements [10]. Accordingly, a sensor fusion method was also studied to determine the driving area with a camera and recognize the exact distance and actual size with a LiDAR [11]. In this study, they identified the part where the ring of the LiDAR was cut off due to a small obstacle in front of the VLP-16 LiDAR. Then, they matched with the semantic segmentation learning results of the front camera image. However, as the calibration of the front camera and LiDAR is also an important research topic, it is difficult to match, and there is a limitation that only objects in the range of the front camera angle can be recognized.

Then, how is it recognizable regardless of object type, satisfying both real-time and performance requirements, and not requiring complex calibration? It is a rule-based recognition technique using LiDAR’s point cloud. This method has been developed since before using deep learning and was mainly studied by judging the ground, which is a plane, and recognizing the part outside the ground as an object.

Recently, many researchers have conducted research to logically extract objects’ characteristics from the point cloud [12]. It is a method of segmenting the area according to the form of a polar coordinate system of the point cloud and extracting the object part by the difference in slope within each area and between each area. It is cumbersome for this method to adjust the slope parameters that determine the characteristics of dividing each area or extracting it as an object. In general, rule-based algorithms have the disadvantage of having many user-definable parameters, on which performance mainly depends. 

However, among rule-based ground removal logics, there is an algorithm that reduces the inconvenience of parameter tuning and is based on raw data from LiDAR that are not separately filtered by RoI [13]. In that paper, parameters were calculated using adaptive ground likelihood estimation and previous results, and a powerful ground segmentation method was proposed by utilizing temporal ground characteristics, regional vertical plane fitting, and noise removal techniques. However, ground clearance alone cannot accurately determine the obstacles autonomous driving must avoid. The point cloud remaining after the ground is removed contains various classes that need to be filtered, such as building walls, curbs, and obstacles. Additionally, lighter and faster algorithms are needed to reduce resources when running in high-speed situations or conjunction with other decision and control algorithms. The algorithm proposed in this study is very simple and fast by utilizing voxels.

Therefore, in this work, we propose a simple small object detection logic that is rule-based but minimizes setup elements and ensures that both performance and real-time requirements are satisfied. After removing noise from the point cloud raw data, the algorithm extracts edges based on the analysis of height value of the point cloud using ring data. It contains the processes that align points with the same ring ID and azimuth to mask object parts according to the rising edge/falling edge. In the last step, we cover edge cases and supplement the algorithm using height and distance indicators. This algorithm was verified by comparing recognition performance and real time in virtual scenarios and actual autonomous driving competition environments with RANSAC [14].

## 2. Background Knowledge

### 2.1. Ring Data of LiDAR

**Figure 1:**
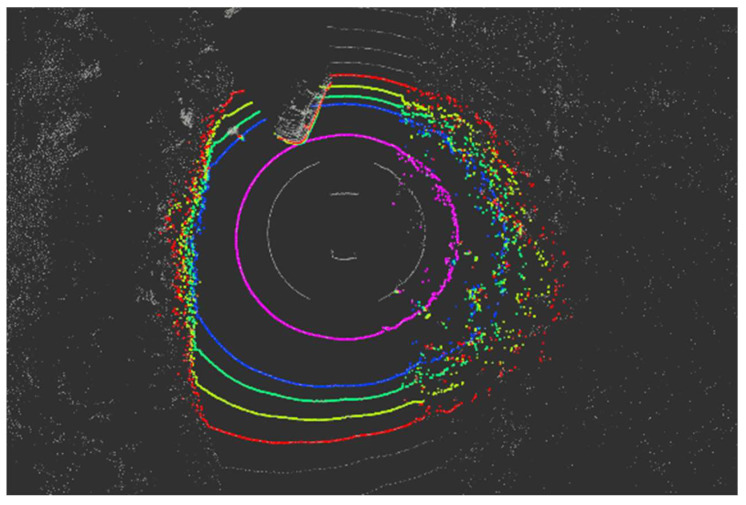
Ring data of point cloud.

Most autonomous vehicles or datasets for deep learning use four kinds of information from the LiDAR point cloud: x, y, z, and intensity. Nevertheless, LiDAR manufacturers provide more information, including ring data [15]. It means the channel ID according to the vertical resolution. Looking at the point shape of the LiDAR beam corresponding to one vertical resolution is described as a ‘ring’; it is called ring data (shown in Figure 1). The ring ID for each point follows the order of small vertical angles (from negative numbers with large absolute values to positive numbers with large absolute values). The total number of IDs equals the number of channels in the LiDAR. For example, the point cloud on a VLP-16 LiDAR has 16 ring IDs ranging from 0 to 15.

### 2.2. Edge Trigger

**Figure 2:**
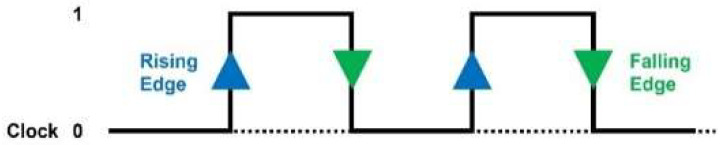
Edge trigger.

When designing logic circuits, the concept of ‘trigger’ is used to detect whether a particular event occurs. The trigger consists of a level trigger and an edge trigger. An edge trigger is an event that occurs when the clock, a state variable, changes. When it changes from 0 to 1, it is a rising edge; when it changes from 1 to 0, it is a falling edge [16], as shown in Figure 2.

### 2.3. Analysis of Object Features in Point Cloud

Before looking at the Ring Edge algorithm in earnest, let us look at the characteristics of objects in the point cloud.

**Figure 3:**
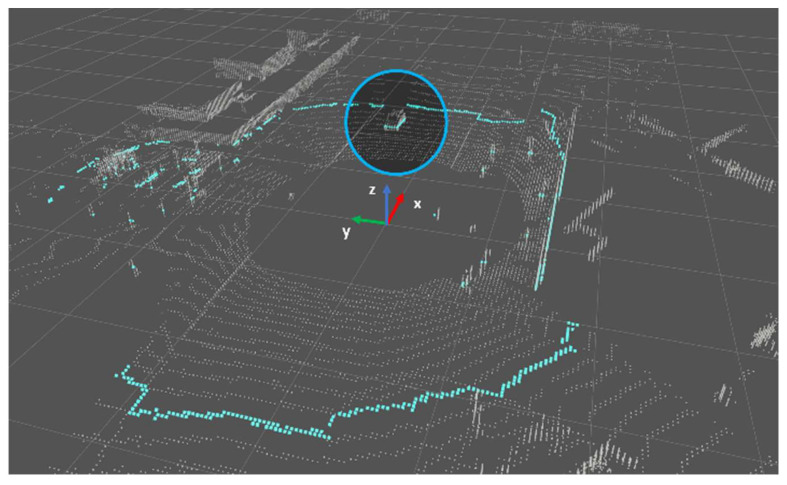
A scene from Waymo Open Dataset.

**Figure 4:**
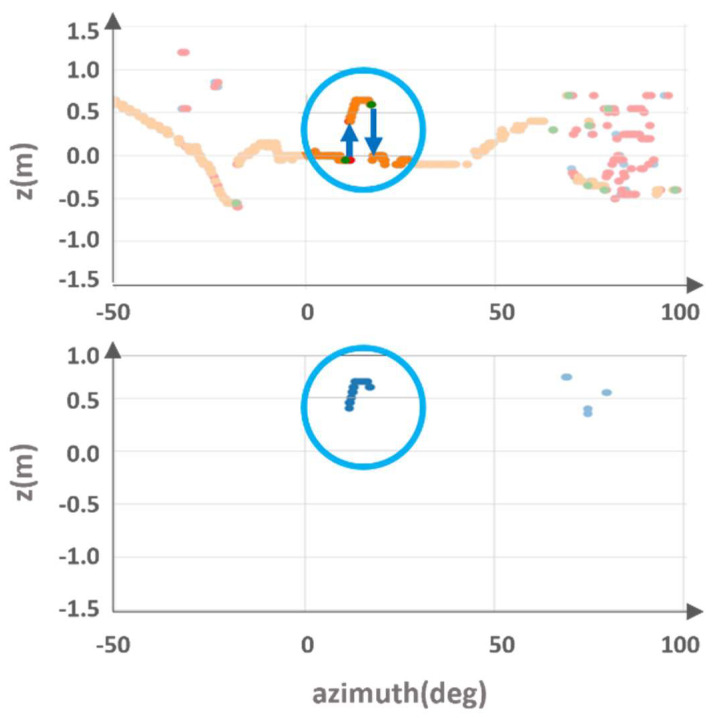
Analysis of z-values from same ring points.

The point cloud is divided into Ring ID groups, and each group is sorted according to azimuth. When looking at the height/distance values, the characteristics displayed by the object in each indicator are as follows. There is a group of points with the same ring ID, as shown in Figure 3 (blue line). The blue circle of Figure 3 represent points corresponding to the object part. The same part is indicated by blue circles in Figure 4. Figure 4 is an enlarged view of the distribution around the object among the z values according to the azimuth of the corresponding group. You can see that the difference in neighboring values before and after the part corresponding to the object increases(inside the blue circle). In particular, the z-value distribution suddenly increases in value with a rising edge at the beginning of the object. It decreases significantly with a falling edge at the end of the object.

## 3. Ring Edge-Triggered Detection Method

This section describes the ring edge-triggered LiDAR detection method and preprocessing for applying the logic.

### 3.1. Noise Filtering

The Ring Edge algorithm proposed in this paper is an algorithm that separates the ground and objects at once after sorting points according to ring ID and azimuth. It is very vulnerable to noise because it is recognized based on when the value difference between aligned neighboring points is large. Accordingly, a part to remove noise was added prior to the full-scale application of the algorithm in the following manner.

First, the edge is found based on a value smaller than the difference between the indicator sizes to be recognized as the edge set as a parameter. Since the found edges are differences between neighboring points in previously sorted points, edge indices can be extracted continuously. Accordingly, the index difference between the extracted edges is calculated, and if it is less than 3, the edges are judged to be noise. If the index difference between edges to be judged as noise is too small, it is difficult to filter out all the noise, and if it is too large, objects with fewer poles or points may also fly away, so some adjustment is necessary.

### 3.2. Object Points Extraction with Ring Edge

The most basic edge trigger-based object point extraction logic is as follows. Edges between neighboring points are extracted based on the parameters ($h_{thres}$) set in the filtered points.

If the z value indicates edge extraction, the part following the rising edge is viewed as an object and masked as 1. The part following the falling edge is viewed as an object only up to the front, and the back part is viewed as ground and is all masked as 0. When the first falling edge is the first falling edge, the front part is viewed as an object, so masking of the front part must also be done.

**Figure 5:**
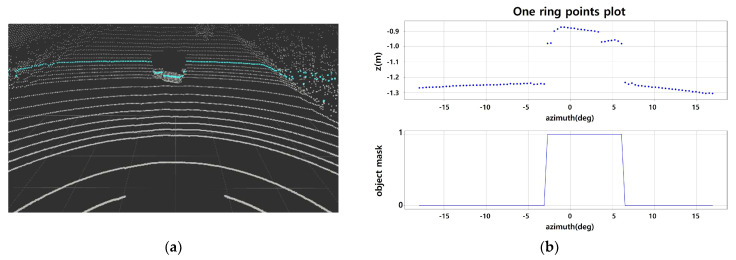
(**a**) Example of one ring of the point cloud; (**b**) 1D state of one ring points; algorithm to detect objects with edges.

Figure 5a is the point cloud scene where a small load is placed in front. If masking is applied to a set of points bounded by the same ring ID, the object part is converted to 1, as shown in Figure 5b. Algorithm 1 of Figure 5 summarizes the basic object extraction logic using Ring Edge from the point cloud from which noise has been removed.
**Algorithm 1. Algorithm to detect objects with edges**  **Input:** Point cloud in multiple areas divided by azimuths  **Output:** Point cloud recognized as obstacle  **for** *each point cloud area in P* **do**    $p_{r}$ *:= p (sorted by ring id)*    **initialize** $p_{result}$    **for** *each ring in R* **do**      $p_{cur}\;$*:=* $p_{r}$*[*$p_{r}.$*ring == each ring]*      *th := arctan(*$p_{cur}$*.y/*$p_{cur}$*.x)*      $p_{cur}\;$*=* $p_{cur}$*(sorted by th)*      $mask_{edge\;}$ *:= list of zeros (size : number of* $p_{cur}$*)*      *diff := difference of z value between neighbor points of*  $p_{cur}$
      *edges := index of*  $p_{cur}$ *where diff >*  $h_{thres}$
      **if** *diff[ first edge ] <* 0 **then**             *// if first edge is falling edge*        $mask_{edge}$*[before first edge] =* 1      **end if**      **for** *each edge in edges* **do**        **if** *diff[each edge] >* 0 **then**        *// if edge is the rising edge*          $mask_{edge}$*[after each edge] =* 1        **else then**                               *// if edge is the falling edge*          $mask_{edge}$*[after each edge] =* 0        **end if**      **end for**      $p_{result}$ *=* $p_{result}$*+*$p_{cur}$*[*$mask_{edge}$*]*    **end for**
**end for**

### 3.3. Covering Edge Cases

However, when using the basic Ring Edge algorithm, there were two edge cases as follows, which were resolved.

**Figure 6:**
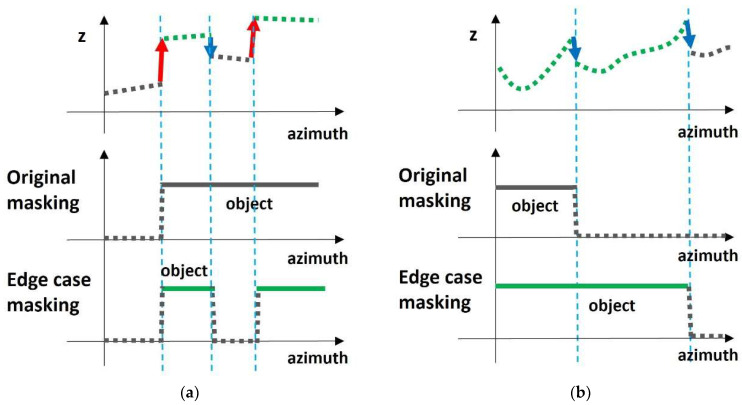
(**a**) Edge case 1: continuous rising edges; (**b**) edge case 2: continuous falling edges.

The first case is when continuous rising edges appear, and the entire area between the two rising edges is masked with an object, resulting in a misrecognition. If you carry this out the original way, all parts behind the rising edge are considered objects, and all parts behind the first rising edge are masked with 1. During this time, whether a falling edge exists is determined based on a value slightly lower than. When extra falling edges exist, misrecognition is improved by masking only the first rising edge to the falling edge with 1, as shown in the example in Figure 6a.

The second case is when continuous falling edges occur, and the object is not recognized because all falling edges are masked with the ground. In this case, if the minimum height between two edges is higher than the minimum height of the part previously masked as an object, the part between the two edges is also considered an object and masked to 1, as shown in the example in Figure 6b.

### 3.4. Results of Processes of Ring Edge

**Figure 7:**
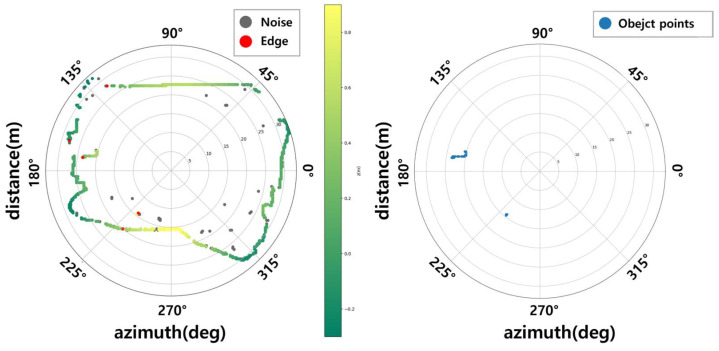
A result of Ring Edge method.

**Figure 8:**
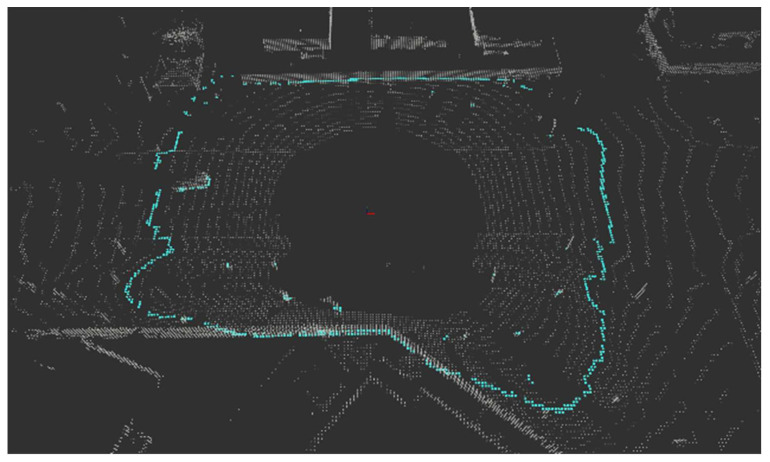
The raw point cloud frame.

Finally, by applying the Ring Edge point extraction logic considering edge cases, the results shown in Figure 7 can be obtained from the input point cloud frame in Figure 8. Figure 8 is another frame from the same sequence in the Waymo Open Dataset. Ring Edge result plots were expressed in BEV form according to azimuth, and raw data from a similar time point was imported for comparison briefly.

### 3.5. Estimation of Bounding Boxes

At points where buildings and walls have been removed to estimate the bounding box based on rules, a clustering process is first necessary. Clustering is dividing points into small sets according to their distribution. Each point is given an ID indicating how many times it has been set. 

**Figure 9:**
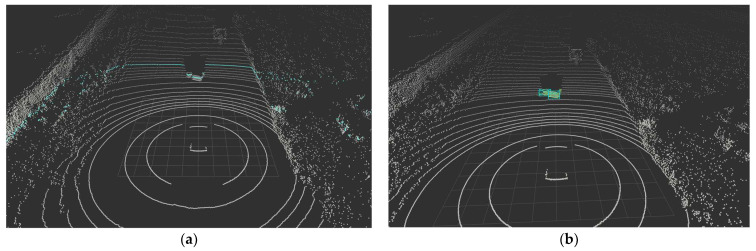
(**a**) Raw point cloud data; (**b**) results of clustering and estimating bounding boxes.

In this paper, we used DBSCAN [17] as the clustering logic. DBSCAN inputs the number of points in a particular space as a parameter. Then, the input parameters become the boundaries that separate each chunk. In this paper, we set it to judge whether at least four points are in a 3 m radius space. Figure 9 clusters the filtered points and estimates the bounding box without rotation based on the size and location of each cluster.

Principal component analysis (PCA [18]) is a dimensionality reduction technique in machine learning and statistics. It transforms high-dimensional data into a lower-dimensional representation by identifying and retaining the most significant features, known as principal components. These components capture the maximum variance in the data, aiding in simplifying its complexity. PCA is valuable for visualization, noise reduction, and speeding up machine learning algorithms. It works by computing eigenvectors and eigenvalues from the covariance matrix of the data, allowing for the selection of the most informative dimensions while minimizing information loss.

This paper uses this PCA algorithm to estimate an object’s heading angle from a clustered point set [19]. We input the point set to find the covariance matrix and the direction vector with the largest variance as eigenvectors. Because the data are three-dimensional points, they return three principal component vectors perpendicular to each other. Among the returned vectors, only two principal component vectors are projected onto the xy-plane. Afterward, the vector’s angle obtained using the tangent becomes the object’s heading angle (yaw).

## 4. Environments of Experiments

### 4.1. Experiments Environment Setting

#### 4.1.1. Open-Source Datasets

**Figure 10:**
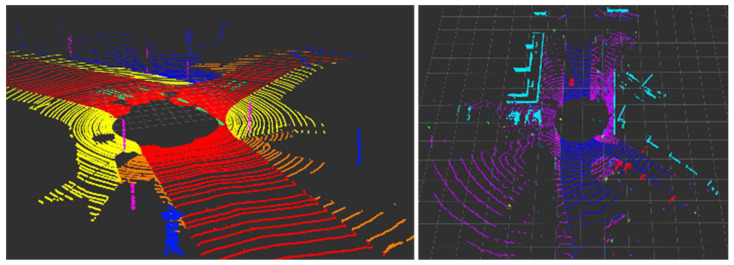
Examples of datasets (**left**: SemanticKITTI; **right**: WOD).

We used SemanticKITTI and Waymo Open Dataset as open-source datasets, which contain 3D semantic segmentation labels. SemanticKITTI [20] is most prevalent in 3D semantic segmentation [21]. It was created using Velodyne HDL-64E and is divided into a total of 28 classes. Because the point cloud in the dataset did not include ring data, which is the critical point of Ring Edge, it must be created. The labeling range of SemanticKITTI is approximately 50 m. Since this is insufficient to confirm the recognition distance, the proposed algorithm was verified using the Waymo Open Dataset, WOD, which has a labeling range of 80 m and is 30 m wider [3]. Figure 10 shows the examples of datasets.

#### 4.1.2. Virtual Driving Environments Setting

The simulation of the virtual environment used IPG’s CarMaker. This simulation can use vehicle models considering vehicle dynamics and customize the location and movement of various obstacles on the road.

**Figure 11:**
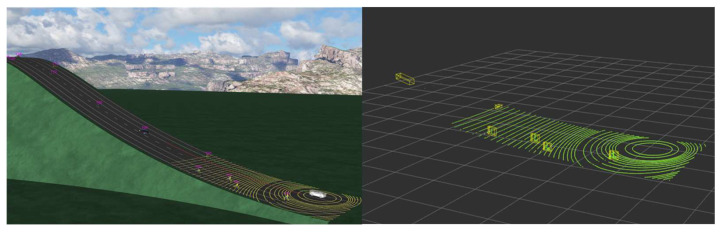
Virtual environment slope scenario.

**Figure 12:**
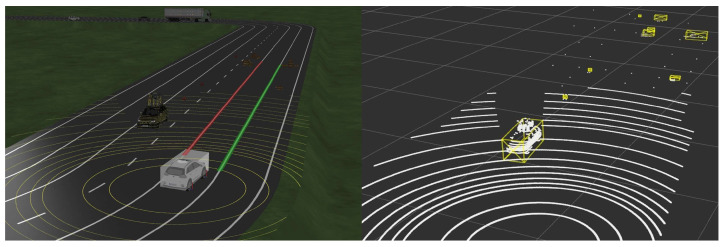
Virtual environment flatland scenario.

As can be seen from Figure 11 and Figure 12, the virtual environment scenario consists of two scenarios: a ramp and a flat road. In each scenario, small obstacles, such as small boxes, garbage bags, and children, and obstacles in the commonly learned deep learning class, such as vehicles, trucks, buses, and pedestrians, are located on the road.

Additionally, a test scenario was created to check the perceived distance according to the size and height of the obstacle. The size of each object used in the test scenario is shown in Table 1.

**Figure 13:**
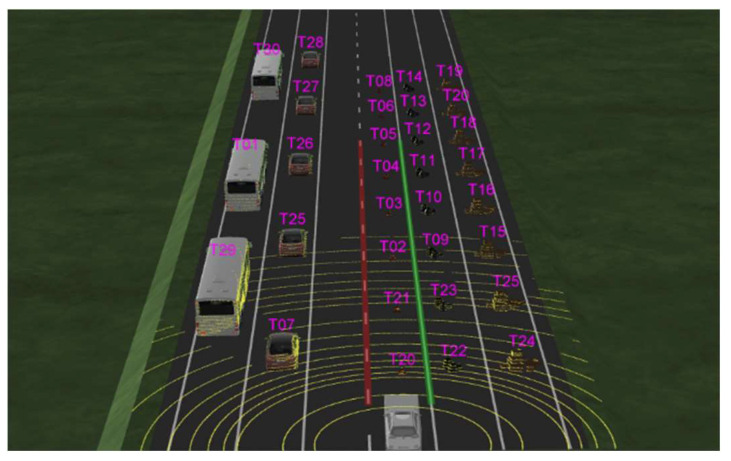
Virtual environment test scenario.

Each object was placed at a certain distance to determine the perceived distance in units of 10 m. In addition, we tried to check the perceived distance according to size and height by arranging objects of various sizes. Figure 13 shows the specific scenario.

#### 4.1.3. Real Driving Environments Setting

**Figure 14:**
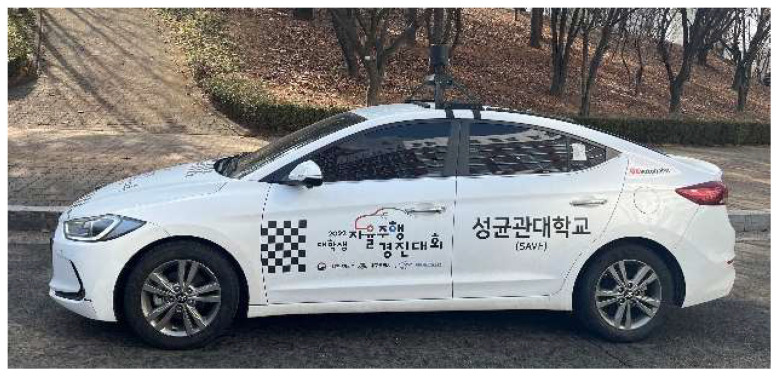
Vehicle of the real environment. (The text written in Korean on the right side of the vehicle indicates the name of the competition and the name of the participating university).

The actual driving environment was set in the 2022 College Student Self-Driving Competition hosted by Daegu Metropolitan City and KIAPI in Korea, as shown in Figure 14. The vehicle used Hyundai Motor’s Avante model, and the LiDAR sensor used Hesai’s Pandar40P model [22] by attaching it to the central roof of the vehicle. The point cloud contains x, y, z, intensity, and ring data elements, and it is a ROS2 middleware-based topic at 20 Hz.

#### 4.1.4. Voxelization Setting

Voxels are an application of the ‘pixel’ concept used in images to point clouds, dividing the three-dimensional space into units based on the point cloud range and voxel size. Depending on the voxel size, multiple points can be contained within one voxel. Additionally, several empty voxels do not contain points due to the point cloud’s sparse features. 

Because multiple points can be expressed by compressing them into a single voxel, voxelizing the point cloud can significantly reduce its size. The size of the point cloud is an essential factor in executing learning networks or logic using the point cloud. For example, PointPillars mentioned in the paper proposing this network that the speed is breakneck at 62 Hz [23]. However, in real situations, the delay when inputting a point cloud obtained from a lidar with 40 channels (Pandar40P) is 60 ms, which cannot maintain 20 Hz. This is because the lidar raw data in the KITTI Dataset [24], the dataset verified in the paper [21], is 64-channel lidar, but only the front part of the ego vehicle is present. So, the number of points is much smaller than that of LiDAR, with 40 channels that are used. Therefore, in learning networks and algorithms, the size of the point cloud is an essential factor that directly affects speed. Therefore, voxelization is necessary to reduce the point cloud’s size primarily.

When voxelizing the WOD, the voxel parameters were set as follows:

range: x(−74.88~74.88), y(−74.88~74.88), z(−2.0~4.0)

voxel size: (x,y,z) = (0.32, 0.32, 0.05)

Before comparing the ground removal performance of Ring Edge when voxelized points were input in earnest, we compared the performance difference before and after voxelizing.

First, regarding speed, when raw point cloud data were input to the Ring Edge, as mentioned earlier, it was 20 ms. When using the PCL library for voxelizing the raw point cloud, 5 ms came out (based on WOD). Then, when the voxel was entered into the Ring Edge, 12 ms came out. It turned out to be faster when inputting the voxel, with a total of 17 ms.

**Figure 15:**
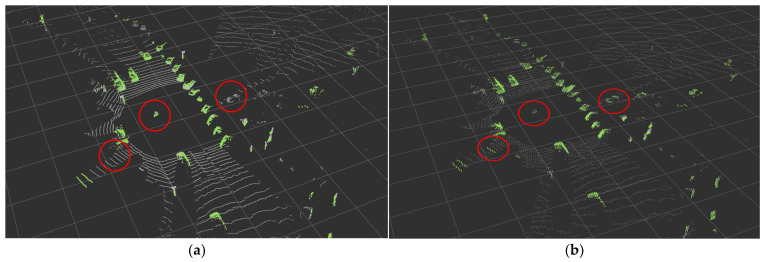
(**a**) Result of the Ring Edge (before voxelizing); (**b**) results of the Ring Edge (after voxelizing).

In terms of performance, some points are lost during the voxel process. As shown in Figure 15, it was confirmed that parts that were well recognized and had no misrecognition in the raw data results showed minor errors in the voxel. However, because there is no significant difference in overall performance except for some, VDR was compared based on voxel.

### 4.2. Evaluation Metrics

#### 4.2.1. F1 Score

The confusion matrix is TP (true positive), where the predicted value is the same number as the correct answer; FP (false positive), which means misrecognition of the predicted value; FN (false negative), which means unrecognized; and TN (true negative), which means neither of the remaining two is correct. F1 score is the harmonic mean value of precision and recall and is a performance index that can consider both misrecognition and non-recognition. It ranges from 0 to 1, with 1 indicating perfect precision and recall balance.

(1) $$precision=\frac{TP}{TP+FP},\;recall=\frac{TP}{TP+FN}$$

(2) $$F1\;score=\frac{2}{\frac{1}{precision}+\frac{1}{recall}}=2\cdot \frac{precision\cdot recall}{precision+recall}$$

#### 4.2.2. Voxel Detection Rate and Point Detection Rate

PDR is originally the pixel detection rate, mainly used in performance verification through camera image pixels [11]. Because the image consists of a dense grid called a ‘pixel’, it is easy to understand that the performance is verified by comparing the pixel area corresponding to the correct answer with the recognized pixel area. To apply this evaluation method to a point cloud, it is usually necessary to convert it into a ‘voxel’ corresponding to a ‘pixel’.

Therefore, all points were converted to voxels through voxelization and entered into Ring Edge. As a result of the ring edge, the VDR (voxel detection rate) indicator was used to determine whether the ground was removed from all points and only the object part was filtered. This is the ratio of the portion of voxels estimated to be an object among the total object ground truth voxels.

(3) $$VDR_{obstacle}=\frac{TPV_{obstacle}}{GTV_{obstacle}}$$

In addition, the rule-based object bounding box estimation logic was verified using the point detection rate, which the pixel detection rate also inspired. In a virtual environment and 3D object detection datasets, they provide accurate bounding box information for each object, and we can easily extract points in this box.

(4) $$PDR_{obstacle}=\frac{TPX_{obstacle}}{GTX_{obstacle}}$$

## 5. Results

### 5.1. Results of Point Extraction Performance

#### 5.1.1. Real-Time Performance

The standard for logic execution speed was the SemanticKITTI point cloud. When comparing logic execution speeds, RANSAC took 15 ms, Ring Edges took 20 ms, and Patchwork++ [13] took 28 ms. Therefore, Ring Edge was confirmed to be slightly slower than RANSAC and faster than Patchwork++.

#### 5.1.2. Detection Performance with F1 Score

Regarding performance, the F1 score was confirmed to be higher for Ring Edge in both semanticKITTI and Waymo Open Dataset. Table 2 and Table 3 provide specific precision and recall values, and the F1 score is calculated accordingly.

**Figure 16:**
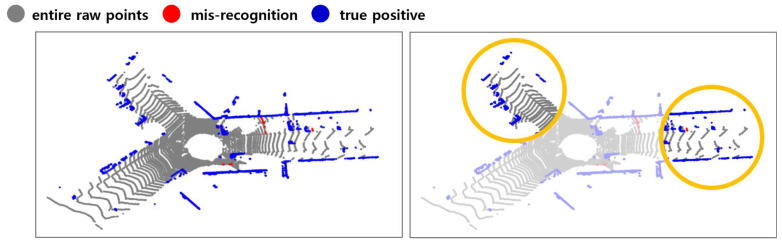
An example of Ring Edge results with SemanticKITTI.

**Figure 17:**
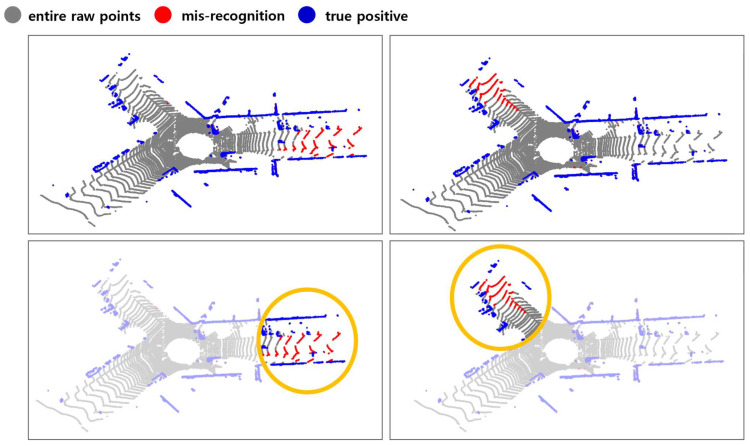
An example of RANSAC results with SemanticKITTI.

Overall, the F1 scores of the proposed algorithms were all higher than RANSAC. Because RANSAC includes randomness in the algorithm, its performance varies each time it is repeated, making it unstable. On the other hand, Ring Edge was confirmed to be more stable and perform better because it always produces the same value. Figure 16 and Figure 17 show RANSAC’s instability and Ring Edge’s excellence.

In particular, the performance of the proposed algorithm was slightly reduced in noisy WOD due to the broader raw data range, while that of RANSAC was noticeably reduced. Because RANSAC is an algorithm that estimates the plane, it cannot estimate correctly if the ground is curved, such as a ramp or a curved road.

#### 5.1.3. Detection Performance with VDR

**Figure 18:**
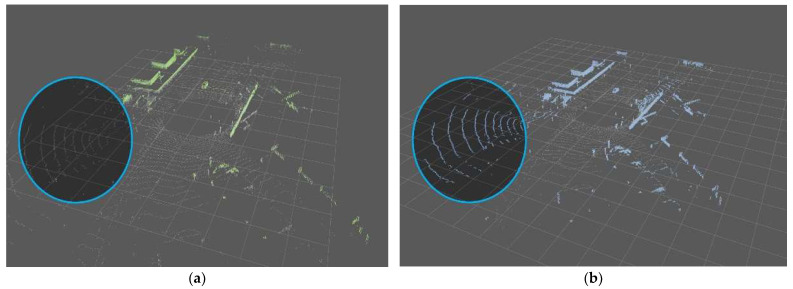
(**a**) Result of recognition of uneven ground (Ring Edge); (**b**) result of recognition of uneven ground (RANSAC).

Next, when the result of converting WOD into voxels was input, the performance was verified using the VDR index. As a result of the comparison, Ring Edge was 0.929, and RANSAC was 0.904, confirming that Ring Edge was superior, like the F1 score index. Looking at Figure 18, Ring Edge removes the ground part well when it is not flat, while RANSAC recognizes it as an object.

### 5.2. Bounding Box Estimation Results

#### 5.2.1. Results with WOD

**Figure 19:**
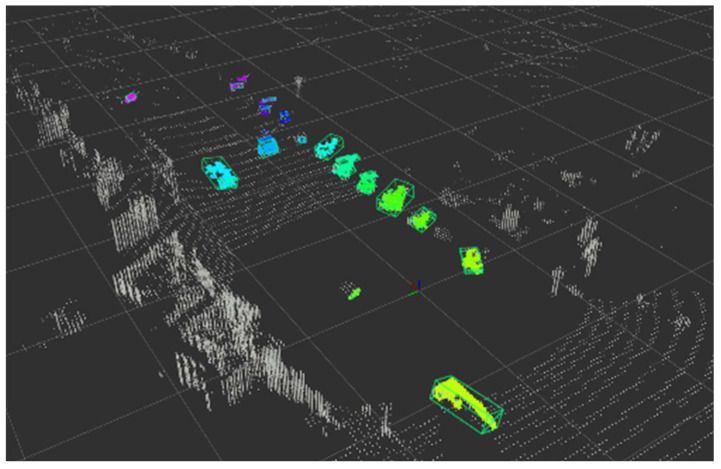
An example of estimated bounding boxes.

As a result of estimating the object bounding box of the voxel-processed WOD based on rules from object partial points from the ring edge, the PDR index was found to be 0.905. The performance was slightly lower than the precision of 94.18% in WOD, which was output when verifying Ring Edge’s performance earlier. This is because object points are lost while removing walls and buildings from points where only the ground was removed. Figure 19 shows the bounding box result estimated from WOD.

#### 5.2.2. Virtual Driving Test Results of Performance

We acted verification in the virtual environment in three scenarios: (1) flatland (mixed objects with small objects) and (2) slope. The point cloud in each scenario was entered in one frame every 0.1 s, and the entire number of points shown in Table 4 represents the total number of points in the bounding box labeled in each frame.

**Figure 20:**
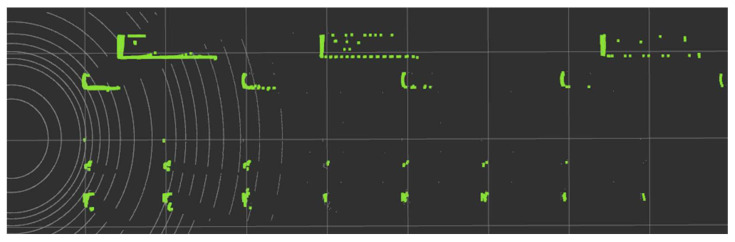
Results of the test scenario.

The results of the test scenario were determined to be recognizable if the points extracted as the Ring Edge results satisfied the minimum number of 3 points of DBSCAN, a rule-based bounding box estimation logic for each object. The results and the distance seen in BEV are shown in Figure 20. Since each division is 10 m, the size and recognition distance for each object can be checked in Table 5.

Therefore, it was confirmed that the recognition distance was longer depending on the object’s height. The tripod was so small at 0.39 m in height that the perceived distance was only 20 m. However, it was confirmed that even when the height was only 0.7 m, the recognition distance increased significantly to 70 m. In the case of cars and buses, the experiment was conducted with the maximum recognition distance in the scenario being 90 m, so the maximum distance for both was 90 m. In the case of buses, they are expected to be recognized even further away due to their large height.

In this scenario, testing was conducted based on Hesai’s Pandar40P LiDAR. Even if a different channel lidar is used, the logic will operate similarly because the group judges it for each ring ID. However, depending on the number of channels, resolution, and LiDAR ray scanning angle, the recognition distance may vary as the number of points captured becomes smaller, even for the same object.

#### 5.2.3. Real Driving Test Results of Performance

There are no points or bounding box correct answers for objects that can be objectively verified when tested in an actual driving environment. It was inevitable to receive and visualize point cloud data in real time and qualitatively evaluate how well they detect. Therefore, we intuitively compared the performance by visualizing the point cloud and the recognition results of each algorithm.

**Figure 21:**
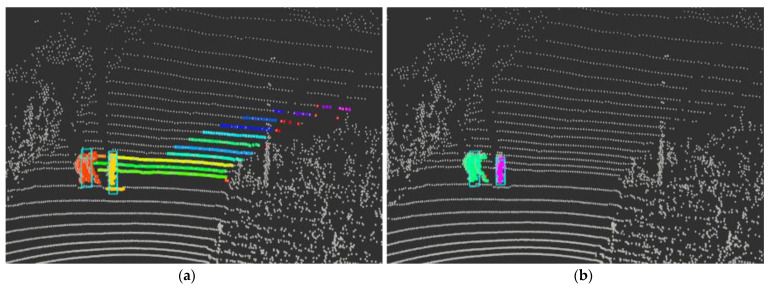
Real driving test results (**a**) RANSAC; (**b**) Ring Edge (ours).

**Figure 22:**
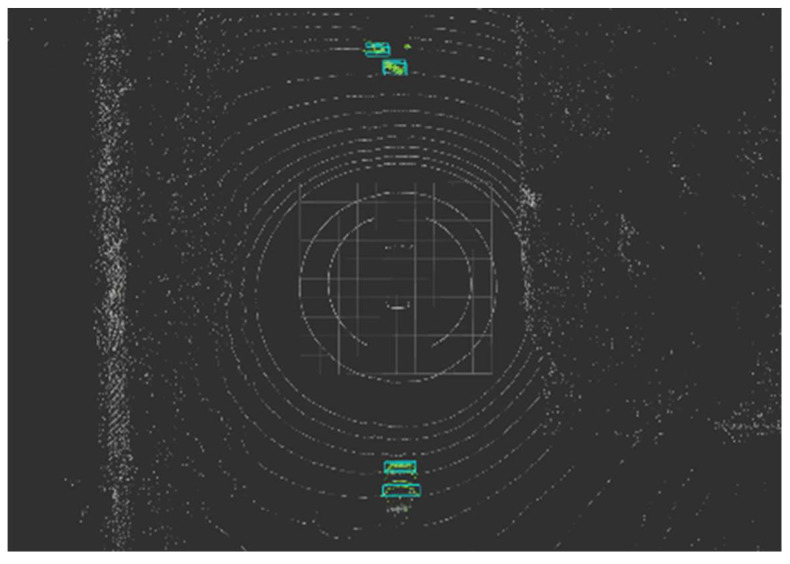
Results of recognizing both front and rear objects (ours).

Figure 21 results from extracting object points in each method when a bent person and a child dummy forward cross the crosswalk. The other color refers to IDs bound to another object the clustering algorithm assigns. Looking at the points extracted as obstacles, the results extracted through the ring edge have much less noise than RANSAC. In addition, Figure 22 shows that both the front load and the competition management vehicle following from the rear were recognized. A complete video related to this can be seen in the following link (https://www.youtube.com/watch?v=lKLvyVGrqDs) (accessed on 15 April 2023) [25].

## 6. Conclusions

This paper proposes a ground removal logic that interprets a group of points aligned with LiDAR Ring ID and azimuth as digital pulses to find edges and mask object parts. In addition, beyond ground removal, the object’s bounding box was estimated using a rule-based algorithm through DBSCAN and principal component analysis.

Ring Edge’s ground removal speed is 20 ms, slightly slower than RANSAC (15 ms) and faster than Patchwork++ (28 ms). In addition, compared to RANSAC based on SemanticKITTI and the Waymo Open Dataset, the F1 score was higher, proving its superiority. The results of estimating the object’s bounding box based on rules from the extracted object voxels were confirmed to be well estimated with a PDR index of over 90% when verified in the Waymo Open Dataset, virtual driving environment, and actual driving environment.

The proposed algorithm guarantees performance and real-time requirements, so it can be applied and used immediately when an autonomous vehicle drives. It was proved by participating in an actual autonomous driving competition and performing a mission to recognize small obstacles by applying this algorithm. Afterward, we will research to learn the bounding box by featuring the object points extracted with this Ring Edge algorithm. Compared to existing end-to-end deep learning methods in terms of speed and performance, we will find the most efficient network and develop logic that can quickly recognize regardless of class.

## Figures and Tables

**Table 1:** The size of each object used in the test scenario.

Kind	Length (m)	Width (m)	Height (m)
Warning tripod	0.16	0.44	0.39
Garbage bags	1.12	1.12	0.7
Boxes	1.46	2.55	1.18
Car	4.83	1.8	1.3
Bus	11.89	2.55	2.65

**Table 2:** Results of performance validation with SemanticKITTI.

	Precision (%)	Recall (%)	F1 Score (%)
Ring Edge	95.59	87.86	91.56
RANSAC	93.09	89.14	91.07

**Table 3:** Results of performance validation with WOD.

	Precision (%)	Recall (%)	F1 Score (%)
Ring Edge	94.18	85.48	89.62
RANSAC	87.02	74.48	80.26

**Table 4:** PDR and the number of points of each method.

Scenario	1 Flatland	2 Slope
True positive	87,417	12,286
False negative	2347	410
Entire points	89,764	12,696
PDR	0.974	0.968

**Table 5:** The detection distance of each object in the test scenario.

Kind	Length (m)	Width (m)	Height (m)	Detection Distance (m)
Warning tripod	0.16	0.44	0.39	20
Garbage bags	1.12	1.12	0.7	70
Boxes	1.46	2.55	1.18	80
Car	4.83	1.8	1.3	90
Bus	11.89	2.55	2.65	90

## Data Availability

Data are contained within the article.
